# Evaluating the Swedish translation of the type 1 diabetes specific health-related quality of life questionnaire in young adults

**DOI:** 10.3389/fcdhc.2025.1720704

**Published:** 2025-12-04

**Authors:** Åsa Carlsund, Sara Olsson, David Rudilla, Ulf Isaksson

**Affiliations:** 1 Department of Nursing, Umeå University, Umea, Sweden; 2 Department of Pulmonology, La Princesa University Hospital, Madrid, Spain

**Keywords:** evaluation, translation, type 1 diabetes, diabetes-specific health-related quality of life, young adults

## Abstract

**Introduction:**

Young adults living with type 1 diabetes face unique challenges as they transition to greater independence, balancing diabetes management in all other dimensions of life. In Sweden, the transfer from pediatric to adult diabetes care at the age of 18 adds to these challenges. This study aimed to translate and evaluate the Swedish version of the T1DAL (Type 1 Diabetes and Life) self-report questionnaire for young adults living with type 1 diabetes.

**Method and Materials:**

The T1DAL questionnaire was translated into Swedish and was completed by 191 young adults aged 18–25 who were registered at a diabetes clinic in three Swedish hospitals. An expert group tested content validity. To determine the number of underlying factors, a parallel analysis (PA) was conducted. The questionnaire’s latent structure was further examined through exploratory factor analysis, in which the items were constrained to a four-factor solution as recommended and found in the original version.

**Results:**

The content validity index of the total score was 0.94. The response distribution analysis revealed the presence of floor or ceiling effects. An EFA with a four-factor solution was conducted, yielding a Model Fit Measure with a χ² of 326.68 and df = 249, resulting in a cmin/df of 1.31, an RMSEA of 0.04, and a TLI of 0.92. Internal consistency was assessed for the subscales suggested by the factor structure, based on the items that loaded onto each factor. Cronbach’s alpha values ranged from 0.75 to 0.89, indicating acceptable to high internal consistency. The four-factor solution explained 45.04% of the total variance.

**Conclusion:**

The Swedish T1DAL questionnaire showed good factorial validity and reliability. The Swedish version requires further testing with potential item reduction; however, it is still expected to be valuable in assessing health-related quality of life among young adults living with type 1 diabetes.

**Clinical implications:**

The Swedish T1DAL questionnaire, particularly its domains related to emotional experiences, managing diabetes effectively, and peer relationships outlines the characteristics of young adulthood and can be used to empower the target group, and is expected to be feasible to implement in clinical practice.

## 1 Introduction

Type 1 Diabetes (T1D) is a lifelong autoimmune disease caused by the destruction of insulin producing beta cells in the pancreas, requiring lifelong insulin therapy (1–3). A main goal in T1D management is to keep blood glucose levels within a target range (4). However, this is a complex task influenced by many factors, including diet, physical activity, and psychosocial conditions (5). Managing T1D requires constant attention and decision-making, which can be physically and emotionally demanding (6).

The transfer from pediatric to adult diabetes care typically occurs when the adolescent living with T1D turns 18 years, in a Swedish context. This shift can be particularly challenging as young adults, usually defined as aged 18 to 25, leave the familiar and often family-involved pediatric care setting and enter a more autonomous adult care environment. Some report feeling unprepared for this transfer, which can further complicate diabetes management (7). The decline of parental involvement in medical visits and decision-making can be both liberating and overwhelming. Effective T1D management extends beyond medical knowledge. The developments during young adulthood involve cognitive, emotional, and social growth, along with the increasing responsibility for one’s health and well-being (8, 9). This period requires gaining the knowledge, skills, and resources necessary to engage in behaviors that promote optimal T1D health outcomes (Boscari & Avogaro, 2021; Kelly et al., 2019; 10). Young adults must also learn to communicate with healthcare providers, ask questions, express concerns, and make informed decisions about their care (Cleal et al., 2022; Van de Velde et al., 2019). Developing these competencies is essential for achieving overall positive health outcomes and maintaining quality of life.

Young adulthood is a period of significant life transitions. For young adults living with T1D, this period presents unique challenges. They must balance diabetes management with new responsibilities and social roles, such as gaining legal autonomy, forming relationships, attending parties, moving from the parental home, managing finances, and navigating academic or professional demands (Gregory et al., 2022; 11; Nettleton et al., 2022; 10). The T1D self-management demands can lead to emotional and physical exhaustion, decreased adherence to treatment, and feelings of isolation (Commissariat et al., 2016; 12; Gregory et al., 2022). Many report difficulties maintaining metabolic control (Gregory et al., 2022; Ingersgaard et al., 2021, 2024) and experience increased stress related to self-management during young adulthood (13–16). Most young adults are in great need of psychosocial support (12).

Considering the complex interplay between medical, psychological, and social factors in T1D management, it is crucial to evaluate health-related quality of life (HRQOL) in this age group. However, there are currently no validated tools in Swedish that measure the psychosocial aspects of T1D self-management among young adults. The Type 1 Diabetes and Life (T1DAL) questionnaire, created by Hilliard et al. (17), is a promising self-report instrument designed specifically for this age group. It can be used in both clinical and research settings but has not yet been translated into Swedish. This study aims to translate and evaluate the Swedish version of the T1DAL questionnaire among young adults (aged 18–25 years) living with T1D. By doing this, we hope to gain a better understanding of diabetes-specific HRQOL in this population and facilitate the development of more tailored interventions and support systems.

## 2 Materials and methods

The process was divided into two phases: Phase 1 involved the instrument translation process, and Phase 2 involved the psychometric analysis.

### 2.1 The T1DAL questionnaire

The T1DAL instrument was developed by Hilliard et al. (17) to assess diabetes-specific health-related quality of life (HRQOL). The version for young adults aged between 18 and 25 includes 27 items, each answered on a 5-point Likert scale (1=No, not at all true, 2=No, not very true, 3=Sometimes true, sometimes not true, 4=Yes, a little true, 5=Yes, very true) based on experiences of the past four weeks. For scoring purposes, each item is transformed to a 0–100 scale: positively worded items are scored as 1 = 0, 2 = 25, 3 = 50, 4 = 75, 5 = 100, and negatively worded items are reverse-scored (marked with an asterisk in **Table 1**). This scoring method follows the original procedure described by Hilliard et al. (17) and allows for a standardized interpretation of HRQOL across items. A total score is calculated by averaging the transformed item scores, yielding a final score ranging from 0 to 100, with higher scores indicating better HRQOL. The instrument assesses four domains: Emotional experiences & daily activities, Handling diabetes well, Peer relationships, and Healthcare experiences. The specific items belonging to each domain are listed in **Table 2**. Hillard et al (17) reported that the instrument takes 5 to 10 minutes to complete.

**Table 1:** Item distribution of the Swedish version of the T1DAL questionnaire. The Swedish version in plain text and the English original version in italics (n=191).

Nummer	Påstående	Nej, stämmer inte alls n (%)	Nej, stämmer inte särskilt bra n (%)	Stämmer ibland och ibland inte n (%)	Ja, stämmer ganska bra n (%)	Ja, stämmer helt n (%)	Missing n (%)	CVI
T_01	Vid behov, är jag bekväm med att be människor utanför min familj om hjälp med min diabetes *I am comfortable asking people outside my family for help with my diabetes if I need a hand*	21 (11.0)	36 (18.8)	59 (30.9)	47 (24.6)	28 (14.7)	0 (0.0)	0.83
T_02*	Jag tycker inte om att prata med mina vänner om min diabetes *I do not like to talk to my friends about diabetes*	67 (35.4)	45 (23.8)	31 (16.4)	34 (18.0)	12 (6.3)	2 (1.0)	1.00
T_03	Mina vänner är hjälpsamma eller stöttande när det gäller min diabetes *My friends are helpful or supportive about my diabetes*	2 (1.1)	9 (4.7)	27 (14.2)	68 (35.8)	84 (44.2)	1 (0.5)	1.00
T_04*	Jag blir frustrerad när människor inte förstår hur det är att leva med diabetes *I get frustrated when people do not understand what it is like to live with diabetes*	27 (14.2)	25 (13.2)	52 (27.4)	40 (21.1)	46 (24.2)	1 (0.5)	1.00
T_05*	Min diabetes stör mitt dejtingliv eller orsakar problem i mitt sexliv *Diabetes interferes with my dating life or causes problems in my sex life*	65 (34.6)	45 (23.9)	48 (25.5)	20 (10.6)	10 (5.3)	3 (1.6)	1.00
T_06	Jag är/skulle vara bekväm med att prata om min diabetes med en partner *I am comfortable talking about diabetes with my romantic/dating partner(s)*	4 (2.1)	4 (2.1)	14 (7.4)	37 (19.6)	130 (68.8)	2 (1.0)	1.00
T_07	Jag känner mig bekväm i att dela min kunskap om diabetes med andra människor *I am comfortable teaching other people about diabetes*	3 (1.6)	6 (3.2)	21 (11.1)	56 (29.6)	103 (54.5)	2 (1.0)	1.00
T_08*	Ibland känns det som om jag är helt ensam om att ha diabetes *Sometimes I feel like I am all alone with diabetes*	36 (19.1)	(21.3)	40 (21.3)	36 (19.1)	36 (19.1)	3 (1.6)	1.00
T_09*	Jag vill inte bli förälder eftersom jag är orolig för att få ett barn med diabetes *I do not want to have children because I am worried about having a child with diabetes*	64 (33.9)	47 (24.9)	41 (21.7)	23 (12.2)	14 (7.4)	2 (1.0)	0.83
T_10	Jag tror att jag kan få en hälsosam graviditet med min diabetes, om jag väljer det *I believe I can have a healthy pregnancy with diabetes, if I want to*	15 (9.1)	23 (14.0)	55 (33.5)	37 (22.6)	34 (20.7)	27 (14.1)	1.00
T_11*	Det är jobbigt att lägga tid på diabetesförberedelser innan jag kan utföra aktiviteter *It is a hassle to* sp*end time preparing for diabetes before I can do activities*	11 (5.8)	20 (10.6)	44 (23.3)	69 (36.5)	45 (23.8)	2 (1.0)	1.00
T_12*	Jag oroar mig för att få lågt blodsocker när jag är fysiskt aktiv *I worry about having a low blood sugar while I am physically active*	7 (3.7)	22 (11.6)	42 (22.1)	54 (28.4)	65 (34.2)	1 (0.5)	1.00
T_13	Jag är rätt bra på att klara av allt jag måste göra kring min diabetes *I am doing okay keeping up with everything I have to do for diabetes*	5 (2.6)	10 (5.3)	23 (12.1)	101 (53.2)	51 (26.8)	1 (0.5)	1.00
T_14*	Jag känner mig tyngd av allt jag måste göra kring min diabetes *I feel overwhelmed with everything I have to do for diabetes*	11 (5.8)	32 (16.8)	62 (32.6)	46 (24.2)	39 (20.5)	1 (0.5)	1.00
T_15	Jag känner mig trygg med att jag kan ta hand om min diabetes när jag ska äta något *I am confident that I can take care of my diabetes when I eat*	2 (1.1)	7 (3.7)	26 (13.7)	70 (36.8)	85 (44.7)	1 (0.5)	0.83
T_16	Även när min diabetes blir svår att hantera, klarar jag det ganska bra *Even when diabetes gets hard to deal with, I handle it pretty well*	1 (0.5)	5 (2.6)	40 (21.2)	93 (49.2)	50 (26.5)	2 (1.0)	0.50
T_17*	På grund av min diabetes är jag inte nöjd med formen/storleken på min kropp *I am not satisfied with the shape or size of my body because of my diabetes*	37 (19.5)	45 (23.7)	43 (22.6)	26 (13.7)	39 (20.5)	1 (0.5)	0.83
T_18*	Det är stressigt att hantera min diabetes när jag äter något *Dealing with diabetes when I eat is stressful*	33 (17.4)	42 (22.1)	65 (34.2)	32 (16.8)	18 (9.5)	1 (0.5)	0.67
T_19*	Jag har svårt att hantera mitt humör eller hur jag agerar när mitt blodsocker är mycket lågt eller mycket högt *I have trouble managing my mood or controlling how I act when my blood sugar is very high or very low*	19 (10.1)	40 (21.3)	42 (22.3)	55 (29.3)	32 (17.0)	3 (1.6)	1.00
T_20*	Jag blir frustrerad över de utmaningar jag för närvarande har med min diabetes *I get frustrated about struggles I am currently having with my diabetes*	17 (9.0)	40 (21.2)	56 (29.6)	43 (22.8)	33 (17.5)	2 (1.0)	1.00
T_21*	Jag oroar mig för hur min framtid kommer att se ut med min diabetes *I worry about what my future will be like with diabetes*	20 (10.6)	30 (15.9)	41 (21.7)	52 (27.5)	46 (24.3)	2 (1.0)	1.00
T_22	Jag kan peppa mig själv när jag känner mig stressad eller upprörd över min diabetes *I am able to cheer myself up when I am feeling stressed or upset about diabetes*	18 (9.6)	41 (21.8)	74 (39.4)	34 (18.1)	21 (11.2)	3 (1.6)	1.00
T_23*	Jag har fått avstå från mycket för att ha råd att hantera min diabetes *I have had to give up a lot to afford my diabetes care*	115 (60.8)	38 (20.1)	20 (10.6)	11 (5.8)	5 (2.6)	2 (1.0)	1.00
T_24	Jag är nöjd med hur mycket av mina diabeteskostnader som täcks av hälso- och sjukvården *I am satisfied with how much of my diabetes expenses are covered by insurance*	4 (2.1)	7 (3.7)	18 (9.5)	37 (19.6)	123 (65.1)	2 (1.0)	1.00
T_25	Jag har en bra relation med mitt diabetesteam *I have a good relationship with my health care team*	5 (2.7)	11 (5.9)	37 (19.7)	67 (35.6)	68 (36.2)	3 (1.6)	0.83
T_26	Jag kan enkelt utnyttja de vårdinsatser jag behöver för min diabetes *I can easily access all of the health care services I need for my diabetes*	3 (1.6)	12 (6.3)	34 (18.0)	78 (41.3)	62 (32.8)	2 (1.0)	1.00
T_27	Jag är nöjd med kvaliteten på den vård jag får från mitt diabetesteam *I am satisfied with the quality of care I receive from my health care team*	2 (1.1)	4 (2.1)	22 (11.6)	76 (40.2)	85 (45.0)	2 (1.0)	1.00

*Items with an asterisk should be reversed.

The response options in English are: No, not at all true; No, not very true; Sometimes true, sometimes not true; Yes, a little true; and finally; Yes, very true.

**Table 2:** Items, factor loadings, total variance explained, Cronbach’s alpha, and McDonald’s omega for the four-factor structure of the Swedish version of the T1DAL questionnaire.

Factor					
Item	Emotional experiences	Peer relations	Health experiences	Handling well	Uniqueness
21	0.77				0.42
14	0.76				0.35
04	0.72				0.55
18	0.7				0.43
20	0.65				0.44
12	0.61				0.68
17	0.59				0.64
08	0.57				0.5
11	0.56				0.58
19	0.52				0.73
09	0.47				0.75
05	0.45				0.62
23	0.34				0.82
10	-0.34				0.82
07		0.79			0.33
06		0.64			0.63
02		-0.63			0.53
01		0.54			0.59
03		0.39			0.59
27			0.86		0.26
25			0.8		0.36
26			0.73		0.44
*24*					0.87
13				0.84	0.33
15				0.62	0.48
16				0.54	0.49
*22*					0.6
Cronbach’s alpha	0.89	0.78	0.83	0.75	
McDonald’s omega	0.89	0.79	0.84	0.76	
% of variance	19.98	9.21	8.72	7.14	

### 2.2 Instrument translation

The first step in Phase 1 was the translation of the English version of the instrument into Swedish. This was done by three of the authors: ÅC, SO, and UI. The translation process involved repeated readings of the original instrument to ensure contextual and conceptual understanding, followed by collaborative refinement to reach consensus. To ensure linguistic and cultural accuracy, the Swedish version was then independently back-translated into English by a native English speaker who had not been involved in the initial translation. The back-translated version was subsequently sent to the original developer of the instrument, Hilliard, for review and approval. Cognitive interviews were conducted to systematically identify and reduce questionnaire errors related to the structure and content of the items in the T1DAL instrument. The purpose was to understand how respondents interpreted each item and chose their responses, provide feedback on the items, and suggest alternative wording. The interviews were conducted online, individually with six individuals aged 18–25 years living with T1D (cf. Boness & Sher, 2020; Castillo-Díaz & Padilla, 2013). Following discussions within the research group, a content validity test (CVI) was conducted, in which the translated questions were sent to a group of six experts, including young adults living with T1D, researchers, and specialist nurses in T1D care. Each expert reviewed each item and assessed whether it was consistent with the instrument’s purpose.

### 2.3 Participants

In Phase 2, the Swedish version of the questionnaire was distributed to young adults aged 18–25 who were listed at a diabetes clinic in three Swedish hospitals from February 18 to August 18, 2025. Inclusion criteria were a diagnosis of T1D for at least one year and an age range of 18 to 25 years. Exclusion criteria included not falling within the age range or being unable to speak or understand intelligible Swedish. Five hospitals chosen at random were contacted in the hope of obtaining assistance with data collection. Of these, three agreed to help with recruitment.

The survey was sent to a total of 559 young adults living with T1D. Of these, 211 participants opened the web-based survey. Of those, three (3) did not give informed consent to participate in the study. Seventeen participants did not answer any items in the questionnaire, resulting in 191 participants for analysis. This resulted in a response rate of 33.5%, which yielded a subject-to item ratio of 7:1. The participants are described in **Table 3**.

**Table 3:** Description of the participants.

Age (mean (SD))	22.98 (1.88)
Sex (n (%))	
Female	114 (59.7)
Male	76 (39.8)
Other	1 ( 0.5)
Type of accommodation (n (%))	
At the parental home	81 (42.6)
Student accommodation	20 (10.5)
Own accommodation	83 (43.7)
Other	6 ( 3.2)
Accommodation site (n (%))	
Urban	150 (79.8)
Rural	31 (16.5)
Sparsely populated area	7 ( 3.7)

### 2.4 Data collection

A brief instruction on how to inform the presumptive participants and how to administer the questionnaire was sent to three diabetes specialist nurses, who agreed to distribute the questionnaire at each clinic. The instruction also included a brief patient information text, along with a direct link to the questionnaire, which all specialist nurses were asked to paste into their respective eHealth systems when contacting individuals who met the inclusion criteria.

Patients were notified by email and then used their Digital ID (i.e., BankID) to access the survey, where they were further informed about the study and the questionnaire. They could then choose to decline participation or proceed and answer the questions. The data were processed in accordance with the GDPR (2016/679), with each response assigned a code. Participants’ informed consent and questionnaire answers were stored securely in Research Electronic Data Capture (RedCap) at Umeå University. The online questionnaire collected demographic information, including age, gender, age at T1D onset, and place of residence. No other identification questions were collected.

### 2.5 Statistical analysis

An item (I-CVI) and scale (S-CVI) content validity index (Polit & Beck, 2006) was calculated based on the questionnaire of the expert group. The S-CVI was calculated as the mean agreement between experts for the whole scale.

An initial analysis involved evaluating the distributional characteristics of the items, including frequency and percentage-based distribution. To identify the number of underlying factors, a parallel analysis (PA) was conducted. Bartlett’s Test of Sphericity was undertaken to assess whether the inter-item correlations were sufficiently strong to justify factor analysis. In parallel, the Kaiser-Meyer-Olkin (KMO) measure was used to evaluate sampling adequacy, determining the overall suitability of the dataset for factor analytic procedures.

The questionnaire’s latent structure was further examined through exploratory factor analysis (Zhu et al.), in which the items were constrained to a four-factor solution as recommended and found in the original version (17). Given that the data were not assumed to follow a normal distribution and inter-factor correlations were anticipated, principal axis factoring was employed as the extraction method, accompanied by direct oblimin rotation (18). Items with factor loadings exceeding 0.3 were retained in the analysis. Model fit was measured using the chi-square (χ2), cmin/df, Tucker-Lewis’s index (TLI), and root mean square error of approximation (RMSEA). Internal consistency was assessed using Cronbach’s alpha and McDonald’s omega. All analysis was performed in Jamovi version 2.6.24.

### 2.6 Ethical considerations

The research for this study complies with Swedish law (19) and the Declaration of Helsinki (20) concerning the four main requirements: information, consent, confidentiality, and utilization of research data. The presumptive participants were asked if they agreed to participate in the study and received written information about the study’s purpose, as well as confirmation that participation was voluntary. They were also told that their medical care would not be affected whether they participated or not. The participants who took part in the cognitive interviews and the content validity test also provided informed consent. All data was stored and analyzed safely in accordance with Umeå University’s regulations and followed a data storage plan.

## 3 Results

### 3.1 Content validity

The CVI ranged from 0.50 to 1.00, with a total score (S-CVI) of 0.94. Two items scored below 0.7, items 16 and 18, with scores of 0.50 and 0.67, respectively. In this study, CVI, along with experts’ statements and cognitive interviews, serves as a complement and is included in the overall assessment of the questionnaire’s validity.

#### 3.1.1 Cognitive interviews

Cognitive interviews was performed to explore how respondents interpreted items, assessed clarity, and identified potential sources of misunderstanding or emotional response. This approach improves item quality and supports content validity by incorporating respondent feedback on relevance, comprehensibility, and interpretability (21, 22). Participants found the items understandable and relevant, although several questions prompted reflection and discussion, revealing nuanced interpretations and areas for improvement. Most items were rated as “very relevant” or “quite relevant,” indicating high subjective relevance. For instance, questions about the role of family support, social interactions, and the emotional burden of T1D were considered important and relatable. Items related to self advocacy and sharing knowledge about T1D received mixed responses – some participants appreciated the chance to reflect on their role in educating others, others felt the responsibility to inform others was not theirs. Another item about diabetes-related preparation for activities was seen as important but potentially vague, with suggestions to specify the types of activities or preparations involved. A few participants mentioned that certain items were especially important or, alternatively, emotionally charged. In those cases, they offered suggestions for additional questions in areas like sexual relationships and pregnancy, and if those topics were emotionally charged, they also suggested rephrasing. Overall, however, participants generally found the questionnaire to be comprehensive and appreciated the chance to reflect on living with T1D.

### 3.2 Distributional properties

The response distribution analysis revealed the presence of floor or ceiling effects—defined as ≥15% of responses clustered at the lowest or highest scale points—for all items except items 1 and 22. Specifically, items 2, 5, 8, 9, 17, 18, and 23 exhibited a floor effect, whereas items 3, 4, 6, 7, 8, 10-17, 19-2, and 24–27 demonstrated ceiling effects. Furthermore, items 8 and 17 exhibited both floor and ceiling effects. One item (item 10) showed a high proportion of missing values (14%).

### 3.3 Exploratory factor analysis

**Figure 1:**
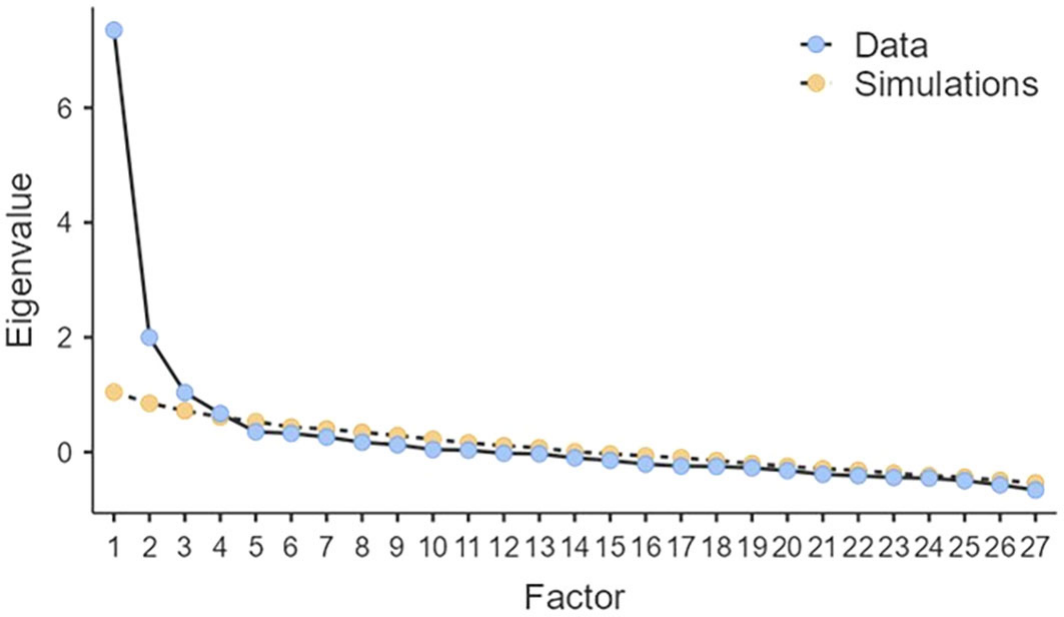
Scree plot of parallel analysis for the Swedish version of the T1DAL questionnaire.

A parallel analysis based on minimum rank factor analysis was conducted to determine the minimum number of factors in the T1DAL questionnaire. According to the analysis, the recommended number of factors was four (**Figure 1**).

Based on the PA, an EFA with a four-factor solution was conducted. Bartlett’s Test of Sphericity showed a p-value of <0.001, and the Kaiser-Meyer-Olkin was overall 0.86. The factors were labelled through a discussion among the research group. Two items did not exceed a loading of 0.30 in any factor, items 22 and 24 (in italics in the table). Model Fit Measures showed a χ² of 326.68 with df = 249, yielding a cmin/df of 1.31. RMSEA showed 0.04 and TLI 0.92.

Internal consistency for the four factors, as estimated by Cronbach’s alpha and McDonald’s omega, was high (0.75 to 0.89 and 0.76 to 0.89, respectively), and the instrument explained 45.04% of the total variance (**Table 2**).

## 4 Discussion

This study aimed to translate and evaluate the Swedish version of the T1DAL questionnaire among young adults aged 18–25 who live with T1D. It demonstrated that the instrument generally has strong content validity, indicating that the Swedish evaluation is reliable and robust, with high agreement among experts on the relevance of items. The translation process involved several steps to ensure meaningful, conceptual, and cultural equivalence. This approach aligns with guidelines emphasizing the involvement of experts and iterative procedures to maintain contextual accuracy (Cruchinho et al., 2024). Cognitive interviewing was employed to evaluate item clarity and participants’ interpretations (cf. 21). In this study, CVI served as a complement to the cognitive interviews and is part of the overall assessment of the questionnaire’s validity, along with evaluation by the expert panel. Overall, the translation process helped ensure the Swedish version of the T1DAL questionnaire was clear and transparent compared to the original, indicating that the instrument could be used in clinical practice and research collaborations.

A commonly accepted threshold for the Scale-Level Content Validity Index (S-CVI) is 0.80. However, Polit and Beck (23) suggest that values as low as 0.70 may be acceptable, whereas Waltz et al. (24) advocate for a more stringent criterion of 0.90 or higher. The Swedish version of the T1DAL achieved an S-CVI of 0.94, indicating a strong result and suggesting that the items effectively capture the intended construct. At the item level, two items (16 and 18) fell below Lynn’s (25) recommended minimum of 0.78. These lower scores may be attributed to challenges in translation and cultural nuances—factors that are critical to consider when adapting instruments across languages and indicate that these items should be removed from the Swedish version of the T1DAL questionnaire. Throughout the adaptation process, efforts were made to ensure conceptual, item-level, and semantic equivalence (26).

Additionally, the response distribution analysis showed that several items had a high proportion of responses at the extreme ends of the scale. While this might initially resemble floor or ceiling effects, it is important to note that such effects are typically evaluated at the scale level rather than the item level (26). In this context, the observed distributions likely reflect the respondents’ strong agreement or disagreement with specific item content, rather than indicating a measurement limitation. For example, items 8 and 17 showed both high and low endorsement, which may reflect diverse experiences rather than poor item design. Nevertheless, these patterns can still be informative for future refinement, particularly in terms of item clarity and relevance. As Liu & Wang (27) suggest, skewed distributions may arise due to sample characteristics, item wording, or response scale design, and should be interpreted with caution.

Furthermore, when an item exhibits both floor and ceiling effects, it may reflect polarization in the population’s experiences—some individuals strongly agree with the statement, while others strongly disagree. This variation could be influenced by factors such as age, gender, duration of diagnosis, or psychosocial support (Simkovic & Traeuble, 2019). Additionally, one item (item 10) exhibited a disproportionately high proportion of missing values. The specific item concerned whether it was possible to have a healthy pregnancy despite living with T1D. This question seemed to be difficult for the participants to answer. It is unclear whether the difficulty lies in the pregnancy itself or if the question has been poorly worded and is therefore difficult to interpret. During the cognitive interviews, both men and women considered the question important and appropriate, as it pertained to family planning. The question was not perceived as unclear or difficult to interpret by those who participated in the cognitive interviews, and they completed this question without delay. The exploratory factor analysis supported a four-factor structure consistent with the original instrument, and the model explained 45% of the total variance. It is also worth adding that the Kaiser criterion suggested a three-factor solution, while the parallel analysis supported four factors. This discrepancy is not unusual, as the Kaiser criterion has been shown to overestimate the number of factors, especially in data sets with many variables or small sample sizes. We therefore prioritized the PA results, which are based on comparisons with simulated data and offer a more conservative and empirically grounded approach to factor retention (28).

Furthermore, the Cronbach’s alpha was surprisingly high for all four domains. This indicates that the internal consistency in all domains between the different items is excellent and that they effectively capture what they are intended to capture. However, factor 1 (Emotional experiences) included 14 items, where two items (10 & 23) loaded low (<0.4). Additionally, two items (items 22 & 24) did not load above 0.3 on any factor. According to Costello and Osborne (18), a factor comprising items with loadings below 0.50 may be regarded as weak and potentially unstable. This suggests that it may be possible to reduce the number of items in the instrument, thereby shortening it and making it easier and quicker to answer, while still capturing the essence of the domain. However, this is something that further studies will need to examine.

One hundred ninety-one people with type 1 diabetes responded to the instrument. This resulted in a response rate of 33.5%, which yielded a subject-to-item ratio of 7:1. This follows established guidelines recommending a subject-to-item ratio of 5:1 to 10:1 (18). The somewhat low response rate may be a result in itself. We believe the questionnaire was neither too long nor too complex, and it did not include items that could be perceived as sensitive. However, some items (e.g., items 23 and 24) were challenging to translate due to cultural and systemic differences between the original and the Swedish context. For instance, questions about insurance coverage for illness-related costs are less relevant in Sweden, where the national healthcare system typically covers such costs. Although cognitive interviews did not reveal any specific issues that would explain non-completion, the use of a web-based format rather than a paper questionnaire may have contributed to the lower response rate. Research consistently shows that web surveys tend to yield lower response rates compared to paper-based surveys, even among younger populations (29, 30). Nevertheless, given the young age of our target group, we expected a preference for digital participation. Another potential factor is the timing of the survey distribution, which occurred shortly before the summer holiday period—a time that may negatively affect response rates. These findings highlight the importance of considering contextual and methodological factors when implementing translated instruments. Future studies may benefit from offering both web and paper formats, extending the data collection period, and enhancing communication strategies to improve engagement and ensure representativeness and generalizability of the findings (31).

The model fit indices from the exploratory factor analysis (TLI and RMSEA) indicated an acceptable fit. Although the chi-squared test yielded a high value with a statistically significant p-value—typically considered undesirable—this result was anticipated due to the test’s sensitivity to sample size (32). Given the relatively small sample in this study (n = 191), a non-significant chi-square was not expected. Notably, the relative chi-square value was 1.31, which falls well within the recommended range of 2.0 to 5.0 (33, 34). In general, other HRQOL instruments often involve assessing diabetes complications and secondary diseases, but T1DAL does not. Complications and secondary diseases most probably impact the overall diabetes HRQOL, and T1DAL not assessing these aspects might be a limitation of the instrument (17). However, international guidelines in diabetes care, such as and White and Cooley (35), promote the importance of highlighting personal strengths in young adults living with T1D. In clinical practice, young adults living with T1D emphasized the importance of including discussions on emotional and psychosocial aspects related to transitions in everyday life, in addition to diabetes-related factors (7).

The factor structure identified in the Swedish version closely mirrors that of the original instrument, suggesting a degree of structural equivalence. Although we used an exploratory factor analysis (EFA), which does not allow formal statistical comparisons across language versions, the similarity in the number and content of factors supports the potential for cross cultural comparability. Future studies could further examine this using confirmatory factor analysis (CFA) and measurement invariance testing.

### 4.1 Limitations

Translating a questionnaire from one language to another involves several challenges, particularly in selecting an appropriate translation methodology. In this study, we employed the widely used forward–backward translation method (36). While this approach is common, it is not without limitations. For instance, Hagell et al. (37) compared forward–backward translation with a dual-panel method and found that the latter resulted in fewer missing items. However, the dual-panel approach is more time-consuming and logistically demanding, and participants may be hesitant to critique one another within a committee setting (36). Given these considerations, we opted for the forward–backward method. Moreover, Maneesriwongul and Dixon (36) emphasize that even when a dual-panel translation is conducted, it should be supplemented with a backtranslation to ensure conceptual and linguistic accuracy. Floor and ceiling effects observed in several items suggest that the instrument’s sensitivity to detect intervention-related changes at the group level may be limited in its current form. Nonetheless, we argue that this does not compromise its practical applicability in clinical settings.

One weakness of our study was the small sample size. The subject-to-item ratio was only 7:1, which is below the recommended 10:1 (18). One reason for the low participation rate may be that the request to participate in the study was sent out just before the summer. Furthermore, the low participation rate also meant that we were unable to perform a confirmatory factor analysis, which could be beneficial in future studies. There is also a risk of selection bias, as we have no data on individuals who chose not to respond to the survey, despite it being sent out to a large population, and we have not been able to compare these groups. However, as this is a psychometric analysis that focuses more on *how* respondents responded to the instrument rather than *what* they responded, this risk is considered to be low.

Another limitation is that we did not conduct a test–retest procedure. As a result, it remains unclear whether observed differences in scores reflect the impact of an intervention or indicate instability in the instrument over time. However, given that health-related quality of life is unlikely to fluctuate significantly over short periods, we consider the instrument suitable for evaluating the potential effects of interventions implemented over a preferably extended duration, and, above all, for clinical use. Nonetheless, further research is warranted to more rigorously establish the instrument’s temporal stability and reliability. The instrument has also not been tested for criterion validity with any similar QOL instrument, which would have been desirable. However, we believe that the T1DAL instrument is very diabetes-specific, and we did not find a similar instrument for comparison. Nevertheless, this is also something that further research and development of the instrument should address.

## 5 Clinical implications

Arnett (38) described the characteristics of emerging adults aged 18 to 29, including the target age group for the T1DAL questionnaire, as exploring their identities, experiencing instabilities in, for example, love, being self-focused, feeling in-between, and being focused on possibilities. We believe that the T1DAL questionnaire (17), particularly the domains related to emotional experiences, handling diabetes well, and peer relationships, frames and addresses the characteristics of emerging adulthood and can be used to empower young adults living with T1D. By using this questionnaire, young adults get opportunities to reflect upon aspects influencing their quality of life, and in discussions with their healthcare providers, they can become more mindful in both strengths and vulnerabilities, leading to empowerment and possible targeted healthcare interventions. In addition, the T1DAL questionnaire is clinically relevant due to taking only approximately five minutes to complete, and is therefore perceived as feasible to implement in clinical practice.

## 6 Conclusion

This study shows good validity and reliability for the Swedish translation of the T1DAL questionnaire. We found, as in the original instrument, four subscales: *Emotional experiences & daily activities, Handling diabetes well, Peer relationships*, and *Healthcare experiences*.

However, the results indicate that there are opportunities for item reduction; therefore, the Swedish T1DAL questionnaire requires further development. Nevertheless, as it stands, it is still expected to be a valuable tool for assessing health-related quality of life among young adults living with T1D.

## Data Availability

The raw data supporting the conclusions of this article will be made available by the authors, without undue reservation.
